# Enhancing gene set enrichment using networks

**DOI:** 10.12688/f1000research.17824.2

**Published:** 2019-07-16

**Authors:** Michael Prummer

**Affiliations:** 1 NEXUS Personalized Health Technologies, ETH Zurich, Zurich, Switzerland; 2 Swiss Institute of Bioinformatics, Zurich, Switzerland

**Keywords:** differential gene expression analysis, gene set analysis, enrichment analysis, network analyis, GSEA

## Abstract

Differential gene expression (DGE) studies often suffer from poor interpretability of their primary results, i.e., thousands of differentially expressed genes. This has led to the introduction of gene set analysis (GSA) methods that aim at identifying interpretable global effects by grouping genes into sets of common context, such as, molecular pathways, biological function or tissue localization. In practice, GSA often results in hundreds of differentially regulated gene sets. Similar to the genes they contain, gene sets are often regulated in a correlative fashion because they share many of their genes or they describe related processes. Using these kind of neighborhood information to construct networks of gene sets allows to identify highly connected sub-networks as well as poorly connected islands or singletons. We show here how topological information and other network features can be used to filter and prioritize gene sets in routine DGE studies. Community detection in combination with automatic labeling and the network representation of gene set clusters further constitute an appealing and intuitive visualization of GSA results. The RICHNET workflow described here does not require human intervention and can thus be conveniently incorporated in automated analysis pipelines.

## Introduction

Interpretation of whole-transcriptome differential expression studies is often difficult because the sheer volume of the differentially expressed genes (DEGs) can be overwhelming. It is common place in designed experiments with more than just a marginal biological effect to find several thousands of differentially expressed genes (DEGs). One way to handle the vast numbers and to identify the biological consequences of gene expression changes is to associate them with overarching processes involving a whole set of genes, such as Gene Ontology (GO) terms or Kyoto Enzyclopedia of Genes and Genomes (KEGG) pathways.

Curated genesets have been designed or discovered for a wide range of common contexts, such as, a biological process, molecular pathway, or tissue localization ^1, 2^. They have been introduced in the past not only to reduce complexity and to improve interpretability but also to increase statistical power by reducing the number of performed tests. As it turns out, this often results in finding hundreds of differentially regulated pathways 1.

As with co-expressed genes, many of the pathways exhibit strong mutual correlation because they contain a large proportion of shared genes which is in turn a result of the fact that many of them describe closely related aspects of an overarching biological theme. Therefore, to further increase interpretability of differential geneset regulation and to capture the global change of a biological phenotype, it would be desirable to identify possibly existing umbrella organizations among genesets.

Networks are ideal to model dependencies, interactions, and similarities among individuals ^3– 5^, be it people, computers, genes, or genesets. The degree of connectivity between them can have an influence on information flow and defines communities or *cliques*, i.e., clusters of highly connected nodes within and infrequent connections between them.

In order to construct a geneset network, a similarity measure is required and can be defined as the fraction of common genes, also called the Jaccard index ^6^. Other ways to measure similarity among genesets include, for instance, coexpression strength as implemented in WGCNA ^7, 8^.

Community detection based on network topology is a standard problem in the analysis of social networks ^9, 10^. Well-established algorithms allow for computationally efficient clustering of genesets and can be used to identify highly connected sub-networks. There is no unique or optimal method available but many options exist. Popular methods to define clusters include the *edge-betweenness* criterion, the *Infomap* or the *Louvain* algorithm ( igraph), as well as hierarchical or kmeans clustering.

Once geneset clusters are defined they can be characterized by their size and connectivity and thus prioritized and ranked. In particular, the clusters can be categorized as singletons, doublets, medium and large or dense and loose clusters.

Network analysis not only allows for detection of clusters and performance of measurements on them, networks are also straightforward and appealing visualizations of similarities among genesets. There are a couple of interactive visualization software tools available, of which Cytoscape is probably the most popular ^11^. In some cases interactivity is useful but the emphasis here is to provide some of Cytoscape’s features without any human intervention for easy integration into automatic analysis pipelines. For instance, automatic labeling of communities using the n most frequent terms was adopted here, similar as in Kucera *et al.* ^12^.

The purpose of this step-by-step workflow is to provide a fully automated and reproducible procedure for downstream analysis and visualization of differential geneset analysis results in R ^13^. The focus is on supporting scientists in result interpretation by bringing order into the list of differentially regulated genesets based on biological rather than pure statistical arguments. The workflow is suitable for any kind of geneset library including new or custom sets and any kind of geneset analysis method.

Starting with differential expression analysis of a model dataset, geneset analysis is performed based on the MSigDB library. A geneset network is constructed to identify isolated genesets (singletons) and geneset pairs (doublets). Larger connected sub-networks are then split into smaller clusters of closely related genesets describing similar processes. The effect of each modification step on the network topology is visually documented in Figure 1– Figure 4. Using the most frequently occurring terms in the geneset names of a cluster, an attempt to automatically assign cluster labels is made. Finally, all labeled clusters of genesets are plotted to provide a one page overview of the results.

## Preparations

The packages required for this workflow provide plotting functions ( ggplot2 and relatives), network functions ( igraph ^14^, etc.), text analytics functions ( wordcloud, etc.) and gene expression analysis functions DESeq2 ^15^, limma ^16^, and org.Hs.eg.db. The Rmarkdown version of this article further requires the Bioconductor package BiocWorkflowTools to be installed.


cranpcks = c("ggplot2","gplots","cowplot","RColorBrewer","knitr","ggrepel","reshape2","kableExtra","igraph","GGally","network","sna","intergraph","wordcloud","tm","SnowballC","BiocManager")
biocpcks = c("DESeq2","limma","org.Hs.eg.db","airway")
tmp = sapply(cranpcks, require, character.only=T, warn.conflicts=F, quietly=T)
toinstall = names(tmp)[!tmp]
if(length(toinstall)>0){
  install.packages(toinstall, quiet=T)
  sapply(toinstall, require, character.only=T, warn.conflicts=F, quietly=T)
} 
tmp = sapply(biocpcks, require, character.only=T, warn.conflicts=F, quietly=T)
toinstall = names(tmp)[!tmp]
if(length(toinstall)>0) {
  BiocManager::install(toinstall)
  sapply(toinstall, require, character.only=T, warn.conflicts=F, quietly=T)
}


In addition to and often based on igraph, several R packages for network visualization are available and described in the form of tutorials ^17, 18^.

### Example data

We are using the popular *airway* data set ^19^ and perform a simple differential expression analysis.


data(airway)
dds =DESeqDataSetFromMatrix(
                        countData = assay(airway),
colData = colData(airway),
design =~cell+dex)
dds$dex =relevel(dds$dex,"untrt")

                        dds =DESeq(dds,betaPrior =T)
res =results(dds,contrast = c("dex","trt","untrt"))


### Mapping Ensembl IDs to ENTREZ IDs

We are using the popular org.Hs.eg.db package based on the UCSC annotation database and keep only genes with a unique mapping.


res$entrezgene =unname(
                        mapIds(org.Hs.eg.db,keys = rownames(res),
column ="ENTREZID",keytype ="ENSEMBL"))
res =subset(res,subset =!is.na(res$entrezgene)& !is.na(res$stat))
res = res[-which(
                        duplicated(res$entrezgene)), ]


### Gene set enrichment analyis

We are using the popular KEGG, Reactome, and Biocarta pathways from the MSigDB gene set library C2. The following chunk guarantees that the gene set library list object is called gset.


url ="http://bioinf.wehi.edu.au/software/MSigDB/human_c2_v5p2.rdata"
temp.space =new.env()
bar =load(url(url), temp.space)
gset =get(bar, temp.space)
rm(temp.space)
gs.libs =sapply(names(gset),function(x)strsplit(x,"_")[[1]][1])
gset = gset[which(gs.libs%in%c("KEGG","REACTOME","BIOCARTA"))]
idx =ids2indices(gene.sets =gset,identifiers =res$entrezgene)


Alternatively, the same result can be obtained using the EGSEAdata experiment package ^19b^.


library(EGSEAdata)
library(EGSEA)
gset = EGSEA::buildMSigDBIdx(entrezIDs = res$entrezgene, 
                             species = "human", 
                             geneSets = "c2", min.size = 3)
idx = gset$c2@idx
gs.libs = sapply(names(idx), function(x) strsplit(x, "_")[[1]][1])
idx = idx[which(gs.libs %in% c("KEGG", "REACTOME", "BIOCARTA"))]


Competitive gene set enrichment analysis is performed using the function camera() from the limma package. We include uni-directional and bi-directional enrichment by using both the test statistics (“up” or “down”) and its modulus (“mixed”) for gene set testing. We limit the following network analysis to gene sets with a *FDR <* 0.05.


dat =cameraPR(res$stat, idx,sort =F)
dat$PValue.Mixed =cameraPR(abs(res$stat), idx,sort =F)$PValue
dat$FDR.Mixed =p.adjust(dat$PValue.Mixed,method ="BH")
dat$name =rownames(dat)

dat$Direction =as.character(dat$Direction)
dat$Direction[dat$FDR>0.05] ="Mixed"
dat$Direction[dat$Direction=="Mixed"&dat$FDR.Mixed>0.05] ="NOT"
dat$Direction =factor(dat$Direction,levels=c("NOT","Up","Down","Mixed"))

idx =which(dat$Direction=="Mixed")
if(length(idx)>0) dat$FDR[idx] = dat$FDR.Mixed[idx]
dat = dat[,-grep("\\.Mixed",names(dat))]
dat = dat[dat$Direction!="NOT", ]
dat$Direction =factor(dat$Direction,levels=c("Up","Down","Mixed"))


Starting from 1077 gene sets, 264 are found to be differentially regulated. Many of them are expected to describe similar processes and to be highly correlated.

## Network construction

We construct a gene set network based on the proportion of common genes as the inverse distance measure. The nodes are gene sets which are connected by edges if the Jaccard index

$$J=\frac{\text{Number}\;\text{of}\;\text{common}\;\text{genes}}{\text{Number}\;\text{of}\;\text{all}\;\text{genes}}$$

is larger than a preset threshold, *J >* 0.2. While this threshold is somewhat arbitrary it has proven to be a reasonable one in many projects. As a guide for finding a reasonable threshold a broad distribution of disjoint cluster sizes is desired. Network analysis does not help if the cutoff is too large (no connections) or too small (all sets are connected with each other). In any case, it is strongly recommended to investigate its effect on the quality of the results.


# only keep gene sets present in the data
id.keep =which(names(gset)%in%dat$name)
gset = gset[id.keep]
# adjacency matrix
m.adj =sapply(gset,function(x)
sapply(gset,function(y)
length(intersect(unlist(x),unlist(y) ))
)
)
diag(m.adj) =0
# Jaccard index matrix
NGenes =sapply(gset, length)
m.union =outer(NGenes, NGenes,"+")-m.adj
m.jacc = m.adj/m.union


The Jaccard matrix, or adjacency matrix, can be conveniently used to construct a network object using the function igraph::graph_from_adjacency_matrix(). In this example geneset, similarity is measured using all member genes irrespective of whether they were detected and present in the data. Alternatively, one could include only genes present in the data depending on whether the current data seem more relevant and trustworthy or the prior information given by the geneset definition. Graphical display is achieved here using ggnet::ggnet2() ( Figure 1).


# choose node colors
palette =brewer.pal(9,"Set1")[c(1,2,9)]
names(palette) =c("Up","Down","Mixed")
# apply cutoff to Jaccard matrix
m.adj1 = m.adj*(m.jacc>0.2)
# construct network object
net =graph_from_adjacency_matrix(m.adj1,"upper",diag =F,weighted =T)
# add vertex features
V(net)$size = dat$NGenes
V(net)$color = palette[dat$Direction]
V(net)$Direction =as.character(dat$Direction)
# plot
ggnet2(net,size =2,color ="Direction",palette =palette,
edge.size =1,edge.color ="#99CC33")


**Figure 1.**
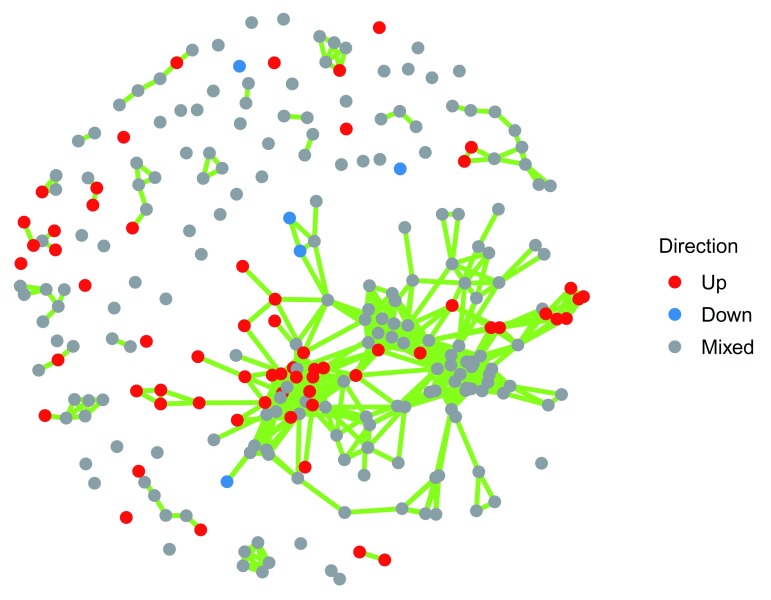
Graphical representation of the initial gene set network. Node colors indicate whether the member genes of a set are predominantly up or down regulated or whether there is no preferential direction (mixed).

## Network modifications

In the following, components of the network for which network analysis does not improve interpretability are identified and put to aside. This includes singletons, i.e., genesets not connected to any other geneset, and doublets, also termed binary systems or dumbbells, i.e., pairs of genesets connected with each other but isolated from the rest.

### Identify singletons


singletons =which(igraph::degree(net)==0)
net1 =delete_vertices(net, singletons)
in.single =which(dat$name%in%V(net)$name[singletons])
tab = dat[in.single, ]
tab$FDR =signif(tab$FDR,2)
tab$name =gsub("_"," ", tab$name)
tab =kable(tab[,c("name","NGenes","Direction","FDR")],
row.names =F,format ="latex",
caption ="List of all singletons, i.e., genesets without
sufficient overlap with any other geneset.")

                        kable_styling(tab,latex_options ="scale_down",font_size =8)


In total, 49 singletons were identified and excluded from further analysis ( Table 1). It is important to note that these genesets, while down-prioritized for the time being, may still be worthwhile investigating later.


ggnet2(net1,size ="size",max_size =4,color =palette[V(net1)$Direction],
size.cut =4,edge.size =1,edge.color ="#99CC33")


**Table 1.** List of all singletons, i.e., genesets without sufficient overlap with any other geneset.

name	NGenes	Direction	FDR
KEGG GLYCOLYSIS GLUCONEOGENESIS	58	Mixed	0.04900
KEGG GLYCINE SERINE AND THREONINE METABOLISM	29	Mixed	0.00020
KEGG ARGININE AND PROLINE METABOLISM	52	Up	0.04100
KEGG GLUTATHIONE METABOLISM	43	Mixed	0.00210
KEGG O GLYCAN BIOSYNTHESIS	26	Mixed	0.01300
KEGG ARACHIDONIC ACID METABOLISM	48	Mixed	0.04300
KEGG NICOTINATE AND NICOTINAMIDE METABOLISM	23	Mixed	0.00290
KEGG CHEMOKINE SIGNALING PATHWAY	160	Mixed	0.02700
KEGG P53 SIGNALING PATHWAY	67	Mixed	0.01400
KEGG APOPTOSIS	78	Mixed	0.02800
KEGG TGF BETA SIGNALING PATHWAY	82	Mixed	0.00140
KEGG ADHERENS JUNCTION	72	Up	0.02900
KEGG LEUKOCYTE TRANSENDOTHELIAL MIGRATION	99	Mixed	0.00360
KEGG PROGESTERONE MEDIATED OOCYTE MATURATION	80	Mixed	0.04900
KEGG ADIPOCYTOKINE SIGNALING PATHWAY	62	Up	0.00180
KEGG PATHOGENIC ESCHERICHIA COLI INFECTION	53	Mixed	0.04000
BIOCARTA AGR PATHWAY	33	Mixed	0.01300
BIOCARTA ATM PATHWAY	20	Mixed	0.00790
BIOCARTA BCELLSURVIVAL PATHWAY	15	Up	0.03400
BIOCARTA LAIR PATHWAY	14	Mixed	0.00910
BIOCARTA EPONFKB PATHWAY	11	Mixed	0.01300
BIOCARTA GABA PATHWAY	6	Mixed	0.04800
BIOCARTA P53HYPOXIA PATHWAY	22	Mixed	0.02600
BIOCARTA EGFR SMRTE PATHWAY	11	Mixed	0.01600
BIOCARTA PPARA PATHWAY	52	Mixed	0.00091
BIOCARTA RAC1 PATHWAY	20	Mixed	0.00440
BIOCARTA NKCELLS PATHWAY	14	Mixed	0.02800
REACTOME METABOLISM OF VITAMINS AND COFACTORS	50	Mixed	0.03100
REACTOME IL 7 SIGNALING	10	Mixed	0.00062
REACTOME SULFUR AMINO ACID METABOLISM	24	Up	0.00530
REACTOME SPHINGOLIPID DE NOVO BIOSYNTHESIS	30	Mixed	0.00970
REACTOME SIGNALING BY HIPPO	20	Mixed	0.00110
REACTOME GASTRIN CREB SIGNALLING PATHWAY VIA PKC AND MAPK	171	Mixed	0.05000
REACTOME PLATELET ADHESION TO EXPOSED COLLAGEN	10	Mixed	0.00910
REACTOME VEGF LIGAND RECEPTOR INTERACTIONS	10	Mixed	0.05000
REACTOME METABOLISM OF AMINO ACIDS AND DERIVATIVES	182	Mixed	0.03100
REACTOME TRANSMISSION ACROSS CHEMICAL SYNAPSES	161	Mixed	0.01900
REACTOME INTEGRATION OF ENERGY METABOLISM	104	Mixed	0.02900
REACTOME CYTOSOLIC TRNA AMINOACYLATION	24	Down	0.03000
REACTOME OLFACTORY SIGNALING PATHWAY	65	Mixed	0.00220
REACTOME SEMA3A PLEXIN REPULSION SIGNALING BY INHIBITING INTEGRIN ADHESION	13	Mixed	0.04000
REACTOME NA CL DEPENDENT NEUROTRANSMITTER TRANSPORTERS	12	Down	0.03700
REACTOME SYNTHESIS AND INTERCONVERSION OF NUCLEOTIDE DI AND TRIPHOSPHATES	18	Up	0.04700
REACTOME ROLE OF DCC IN REGULATING APOPTOSIS	10	Mixed	0.00420
REACTOME NETRIN1 SIGNALING	35	Mixed	0.00940
REACTOME NEPHRIN INTERACTIONS	17	Up	0.03000
REACTOME RAP1 SIGNALLING	15	Mixed	0.00900
REACTOME ETHANOL OXIDATION	10	Up	0.00012
REACTOME HORMONE SENSITIVE LIPASE HSL MEDIATED TRIACYLGLYCEROL HYDROLYSIS	11	Mixed	0.01000

**Figure 2.**
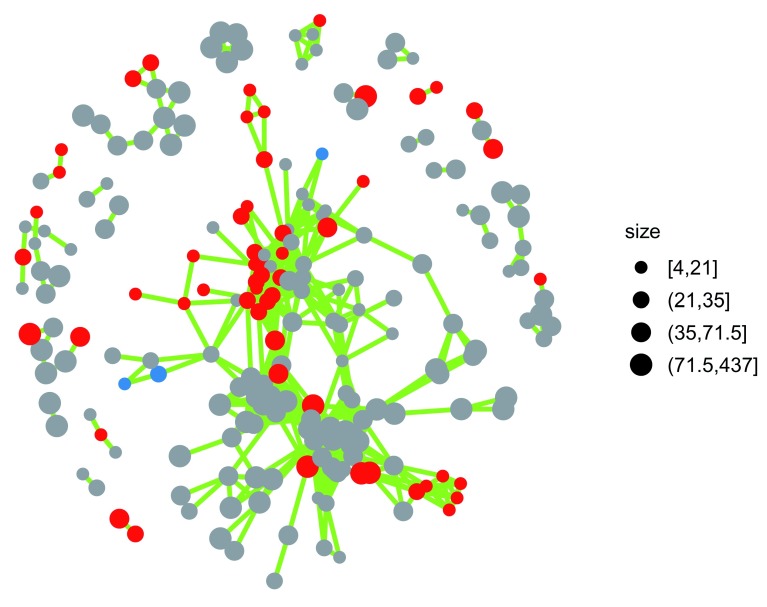
Gene set network with singletons removed. The color scheme is the same as above. The node size corresponds to the number of genes in a set.

Figure 2 shows the remaining network clusters, with the size of the nodes representing the number of genes in the set.

### Identify binary systems (2 sets)

Next we also want to separate clusters with less than 3 gene sets. To do so, we separate disjoint subnets as individual objects, count their members, and delete all vertices belonging to clusters of size smaller than 3.


clu1 = igraph::components(net1)
clu.lt3 =which(sizes(clu1)<3)
v.clu.lt3 =which(clu1$membership%in%clu.lt3)
net2 =delete_vertices(net1, v.clu.lt3)
clu2 = igraph::components(net2)
in.clu.lt3 =which(dat$name%in%V(net1)$name[v.clu.lt3])
tab = dat[in.clu.lt3, ]
tab$FDR =signif(tab$FDR,2)
cludp = clu1$membership[v.clu.lt3]
cludp =data.frame(name = names(cludp),id = as.numeric(cludp))
tab =merge(tab,cludp)
tab$name =gsub("_"," ",tab$name)
tab =kable(tab[order(tab$id),c("id","name","NGenes","Direction","FDR")],
row.names=F,format ="latex",
caption ="List of binary clusters as indicated by the id column.")

                        kable_styling(tab,latex_options ="scale_down",font_size =8)



In Table 2, consecutively listed gene sets with the same *id* belong to the same binary cluster. Often these are gene sets from different libraries describing the same biological process or phenotype. In total, 16 binary clusters were identified, for which network analysis would not be useful.


set.seed(16)
nodecol =colorRampPalette(brewer.pal(9,"Set1")[sample(
                        9)])(max(clu2$membership))
ggnet2(net2,size ="size",max_size =4,color =nodecol[clu2$membership],
size.cut =4,edge.size =1,edge.color ="grey")



**Figure 3.**
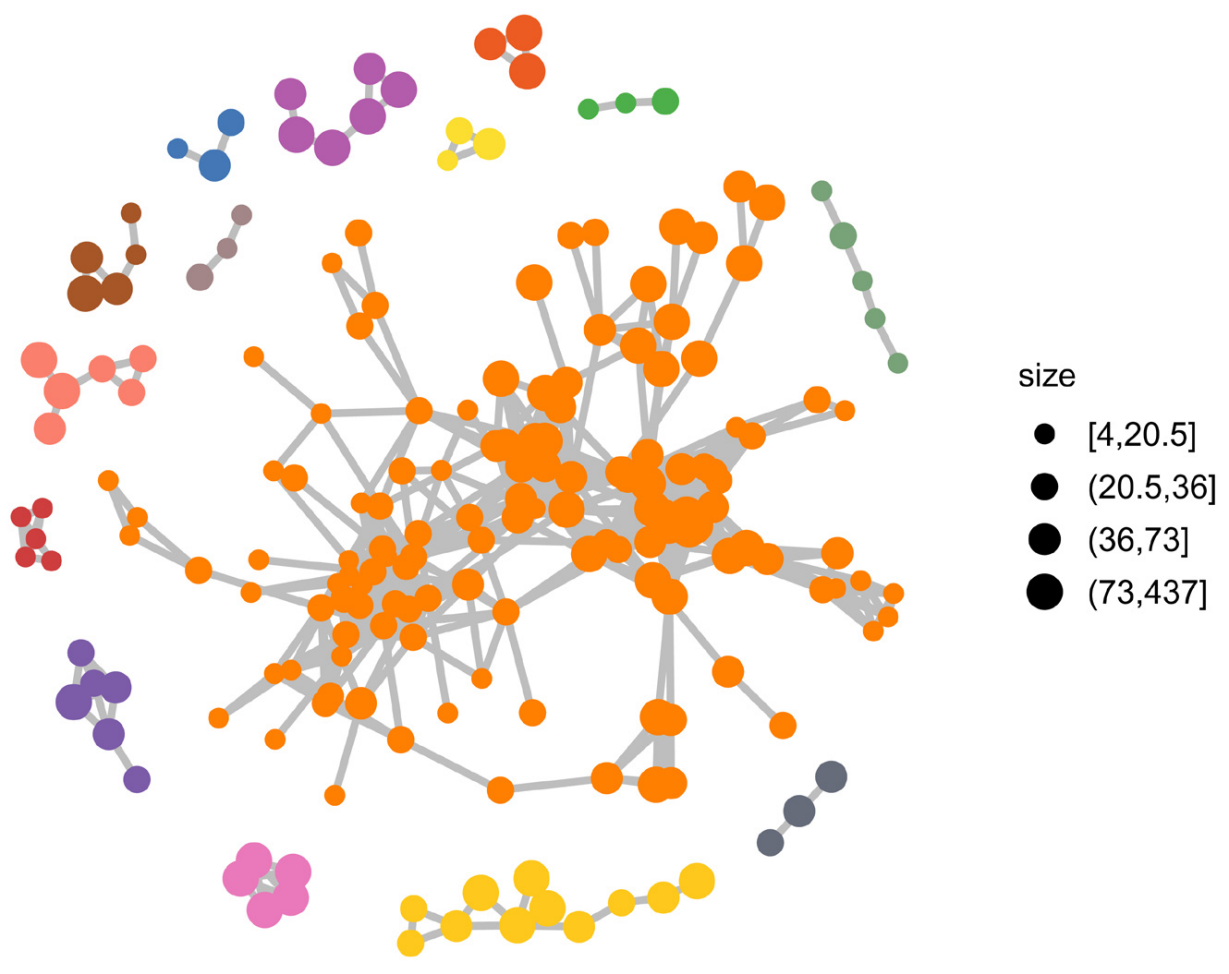
Gene set network with singletons and binary clusters removed. Colored according to disjoint subnetworks.

**Table 2.** List of binary clusters as indicated by the id column.

id	name	NGenes	Direction	FDR
3	KEGG ALANINE ASPARTATE AND GLUTAMATE METABOLISM	28	Mixed	4.9e-03
3	REACTOME AMINO ACID SYNTHESIS AND INTERCONVERSION TRANSAMINATION	16	Mixed	3.6e-04
6	KEGG INOSITOL PHOSPHATE METABOLISM	49	Mixed	2.0e-04
6	KEGG PHOSPHATIDYLINOSITOL SIGNALING SYSTEM	69	Mixed	1.5e-04
17	BIOCARTA ARF PATHWAY	16	Mixed	1.2e-02
17	BIOCARTA CTCF PATHWAY	22	Mixed	3.8e-04
18	REACTOME PLATELET ACTIVATION SIGNALING AND AGGREGATION	178	Mixed	2.9e-03
18	REACTOME RESPONSE TO ELEVATED PLATELET CYTOSOLIC CA2	72	Mixed	3.9e-02
19	REACTOME NEUROTRANSMITTER RELEASE CYCLE	28	Up	1.4e-02
19	REACTOME NOREPINEPHRINE NEUROTRANSMITTER RELEASE CYCLE	10	Up	4.5e-02
20	REACTOME AMINO ACID AND OLIGOPEPTIDE SLC TRANSPORTERS	40	Mixed	2.0e-02
20	REACTOME AMINO ACID TRANSPORT ACROSS THE PLASMA MEMBRANE	29	Mixed	8.7e-03
22	REACTOME MUSCLE CONTRACTION	42	Up	3.4e-05
22	REACTOME SMOOTH MUSCLE CONTRACTION	23	Up	0.0e+00
23	REACTOME ACTIVATION OF GENES BY ATF4	24	Mixed	7.8e-03
23	REACTOME PERK REGULATED GENE EXPRESSION	27	Mixed	2.0e-02

Without singletons and binary clusters, we are left with larger disjoint subnets ( Figure 3).

### Detect communities (sub-networks)

The larger disjoint clusters may consist of so-called *communities*, i.e., sub-networks of highly inter-connected nodes that stick together by only one or a few edges. We are using the popular *edge betweenness* property to identify these community-connecting edges and remove them in order to split large clusters into smaller ones.


net2 =delete_edge_attr(net2,"weight")
clu3 =cluster_edge_betweenness(net2)
# delete edges between communities
net3 =delete_edges(net2,which(as.vector(crossing(clu3, net2))) )
# remove clusters of size <3
small_cluster_ids =which(sizes(clu3)<3)
small_cl_v =which(clu3$membership%in%small_cluster_ids)
net3 =delete_vertices(net3, small_cl_v)

clu3 = igraph::components(net3)
nodecol =c(brewer.pal(9,"Paired"),brewer.pal(9,"Set3") )
nodecol =colorRampPalette(nodecol)(max(clu3$membership))

ggnet2(net3,size =0,color =nodecol[clu3$membership],
edge.size =1.0,edge.color ="grey")+
geom_point(size =2,color ="black")+
geom_point(aes(color =color),size =1)


**Figure 4.**
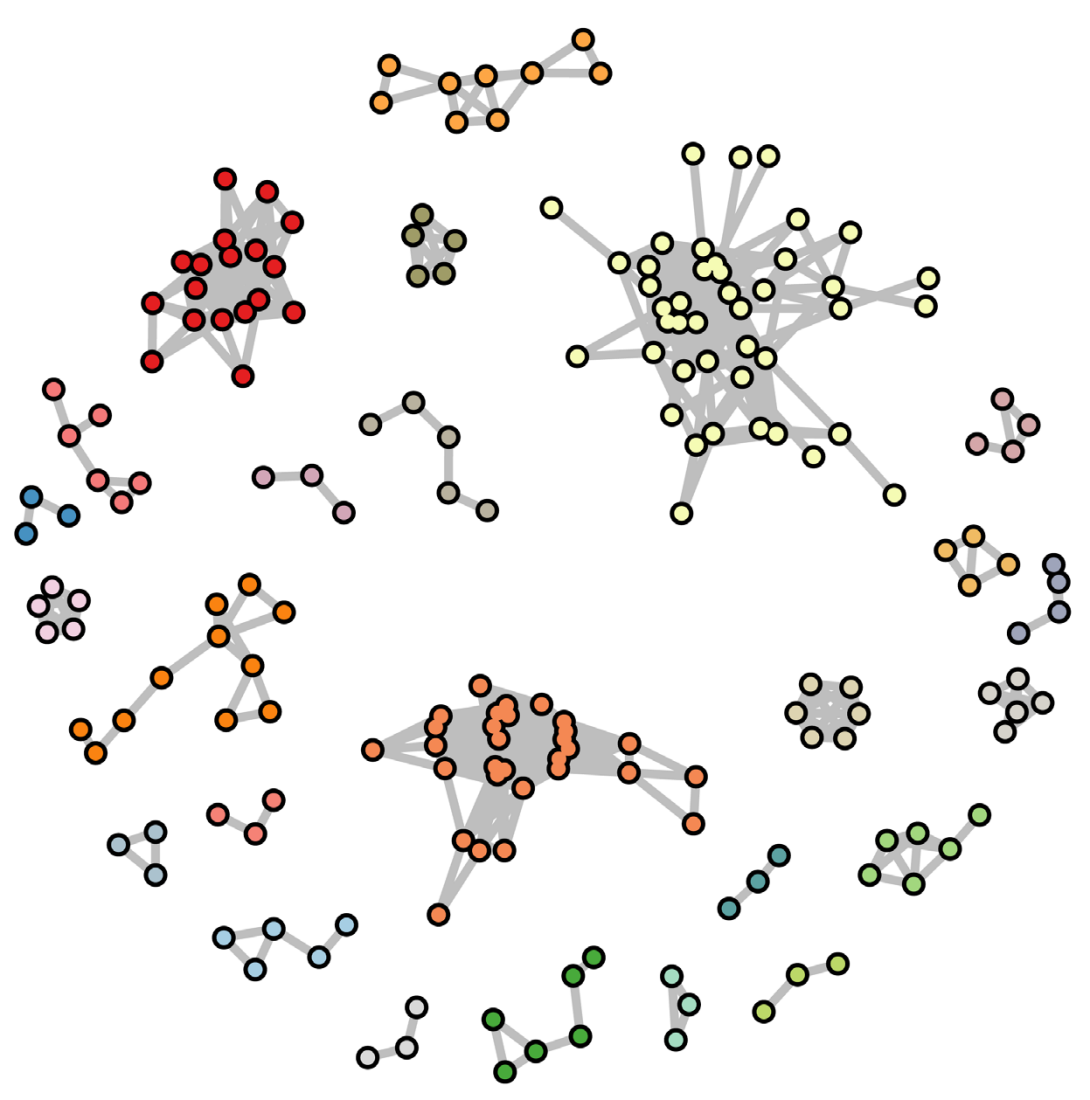
Disjoint clusters after community detection and splitting.

The result of this network-based clustering is shown in Figure 4

## Automatic annotation of gene set clusters

In analogy to the popular interactive network visualization tool *cytoscape* ^12^, we attempt to generate automatic labels for gene set clusters. Gene set names are split into individual words and counted within each cluster. The four most frequent terms occurring at least twice are used as labels. The function clust_head() is defined for this purpose and contains an exclusion list of words not used.


t.rW =c("cell","process","regulation","negative","positive","signaling",
          
                    "response","stimulus","signal","activity","protein","involved",
          
                    "component","level","effector","event","projection","organismal",
          
                    "cellular","modification","pathway","mediated","dependent",
          
                    "organization","group","target","biocarta","kegg","reactome")
clust_head =function(x){
txt =unlist(strsplit(x,"_"))
txt =Corpus(VectorSource(txt))
txt =tm_map(txt, PlainTextDocument)
txt =tm_map(txt, removePunctuation)
txt =tm_map(txt, removeNumbers)
txt =tm_map(txt,content_transformer(tolower))
txt =tm_map(txt, removeWords,c(t.rW,stopwords("english")))
tdm =TermDocumentMatrix(txt)
m =as.matrix(tdm)
word_freqs =sort(rowSums(m),decreasing=TRUE)
word_freqs = word_freqs[word_freqs>1]
word_freqs =paste(
                    names(word_freqs)[
                    1:4],collapse=" ")
gsub("[[:space:]]?NA[[:space:]]?","", word_freqs)
}



### Lattice of annotated networks

**Figure 5.**
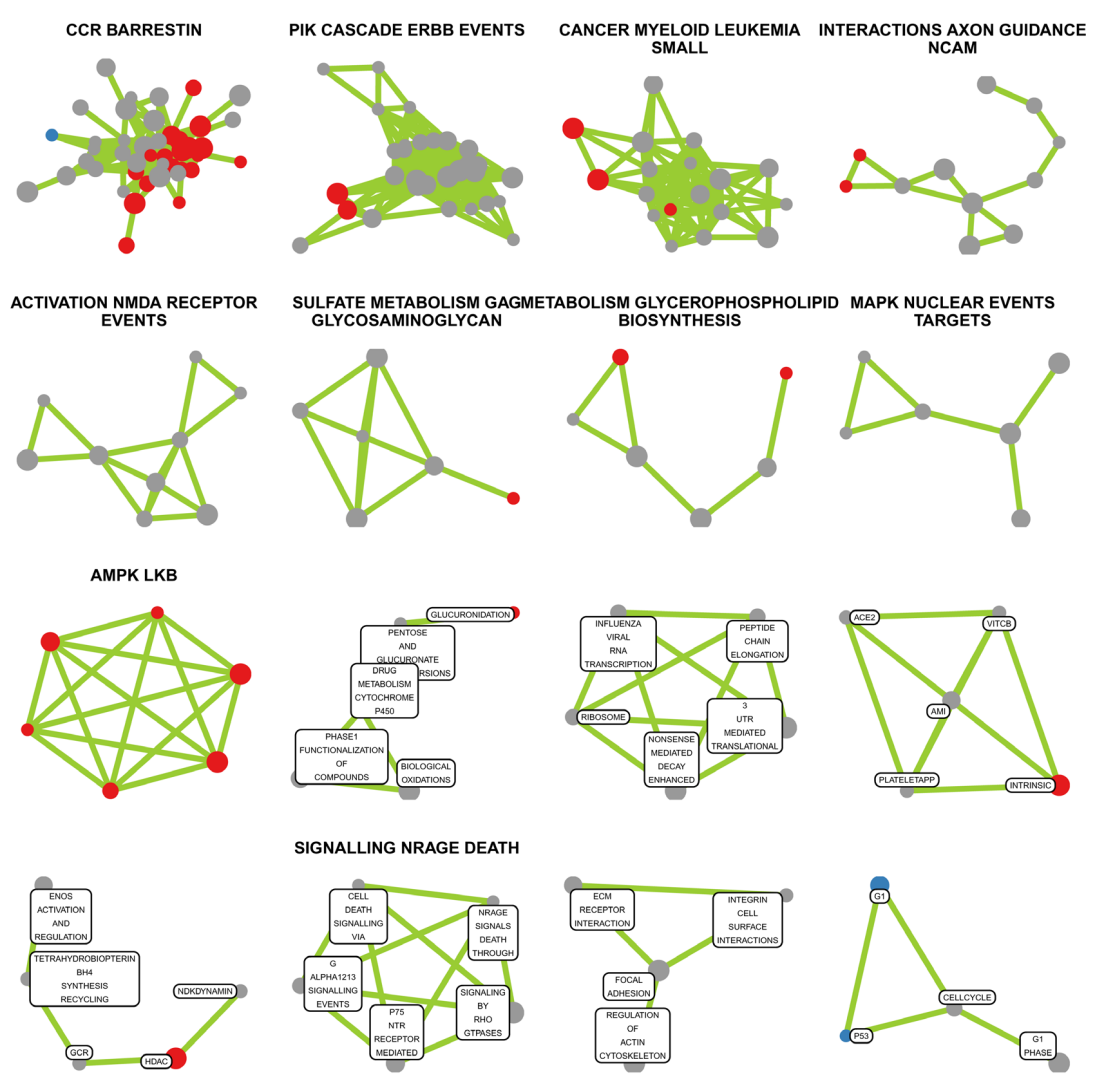
Geneset cluster with machine-generated titles. Only the first 16 connected subnets are shown. Geneset labels are omitted for clusters with more than 5 members.

There are many possibilities to visualize geneset clusters and often a compromise between information content and crowding has to be found. Here, we are producing a lattice of network plots, one for each sub-net, with the automatic annotation as title ( Figure 5). We begin by generating the cluster titles using the clust_head() function followed by cleaning up and ordering by cluster size.


clust =data.frame(cl =clu3$membership)
rownames(clust) =names(V(net3))
# generate cluster titles
cl3.lab.txt =as.character(tapply(rownames(clust), clust$cl, clust_head))
# remove NAs
cl3.lab.txt =gsub("[[:space:]]?NA[[:space:]]?","", cl3.lab.txt)
clu3 = igraph::components(net3)
clu.order =order(clu3$csize,decreasing =T)
clu3$mem =match(clu3$membership, clu.order)


Then we generate a list of ggplot objects, one for each cluster or sub-net. For smaller sub-nets, the nodes are labelled with the first 4 words of their names; the first word was removed before as it is usually the name of the geneset library. For larger sub-nets, this is not feasible without overprinting. Titles are missing if none of the words from the geneset names occurred more than once. This may be indicative for a semantically mixed cluster or for sparse prior knowledge.


# generate a list of ggplots
g =list(max(clu3$membership))
set.seed(7042016)
for(iiin1:max(clu3$membership)) {
subgf =induced_subgraph(net3,which(clu3$mem==ii))
# generate titles with one optional line break
title =substr(toupper(cl3.lab.txt[clu.order][ii]),1,60)
if(nchar(title)>25) {
title =sub("(^.{10,30})[[:space:]]","\\1\\\n", title)
}
# generate node labels using word 2-5 of the geneset name
v.label =names(V(subgf))
v.label =lapply(v.label,function(x)strsplit(x,"_")[[1]])
v.label =sapply(v.label,function(x)paste(x[2:min(5,length(x))],collapse ="_"))
# clean up geneset names
v.label =gsub("_PATHWAY","", v.label)
v.label =gsub("_SIGNALING","", v.label)
# introduce line breaks
v.label =gsub("_","\n", v.label)
# remove node labels for large clusters
if(length(v.label)> 5) v.label =rep(NA,length(v.label))
g[[ii]] =ggnet2(subgf,edge.size =1, 
                        edge.color ="#99CC33",
                     
                        label =F,size=V(subgf)$size,max_size =3,
                     
                        size.cut =4, 
                        color =palette[V(subgf)$Direction])+
theme(
                        legend.position="none", 
                        plot.title = element_text(
                        size=6),
           
                        panel.grid = element_blank())+
geom_label_repel(
                        label =v.label,size=1.2,
                       
                        box.padding =0.1, 
                        label.padding =0.1) 
                        +
ggtitle(title) }




nr.cols =min(4,
                        max(clu3$membership))
nr.rows =ceiling(
                        max(clu3$membership)/nr.cols)
width =sapply(g,function(x)nrow(x$data))
grid.arrange =getFromNamespace("grid.arrange", 
                        asNamespace(
                        "gridExtra"))
grid.arrange(
                        grobs =g[seq(
                        16)],ncol =nr.cols)



## Discussion

We have presented an automated workflow based on a small number of R packages for prioritization and visualization of gene set analysis results using networks, which we call RICHNET. We demonstrated how community detection facilitates categorization of differentially regulated gene sets into singletons and clusters of different size ranges. Automated label generation allowed to associate these clusters with biological themes or processes of which the member gene sets are part of.

The RICHNET workflow could be altered or extended quite naturally in a number of ways but the version presented here is the one we typically apply in our research service projects. One advantage over other approaches is that it does not depend on a particular geneset library or geneset analysis method. Any means of selecting genesets of interest can be used. Specific hierarchically constructed genesets, such as GO terms, would offer a straightforward way to arrive at a more global process description using higher levels in their tree structure. A second advantage is that it does not depend on the existence of a good quality gene or protein interaction network for the particular organism or disease state which is often not feasible. Only very few genesets are network-based (e.g. KEGG pathways) and would thus offer a straight-forward way to use an *a priori* network topology. Thirdly, similar as in reference 8, a geneset similarity network could be constructed in the form of a co-enrichment network from GSVA enrichment scores ^20^ using weighted co-expression network analysis (WGCNA) ^7^. However, this approach relies on a relatively large sample size whereas the sample size requirement of RICHNET is not more than the GSA it relies on.

As an alternative to the networks of genesets described here, networks of genes could be created in a reciprocal way. The underlying similarity metric between genes could be defined as the proportion of common genesets among all genesets they are part of. This approach would be equivalent to a STRING-DB network with “databases” as the only interaction allowed ^21^.

One possible future extension of the RICHNET workflow could be the introduction of a consensus similarity metric from multiple initial networks and different community detection or cluster algorithms to improve stability against noise. A second avenue forward could be the introduction of interactive graphics in 2D or 3D ^17^ to allow moving, pulling, rotation or zoom and display of node specific or edge specific information.

Some may argue in favor of encapsulating the RICHNET workflow in an R or Bioconductor package. However, it is our strong believe that for the sake of transparency and given the straightforward nature of the code it serves better to publish it openly. This way we encourage the users to adapt it to their specific requirements, to improve and expand on it.

## Data availability

The data used in this workflow is included in the *airway* R-package ^19^.

## Software availability

**The R markdown file for this workflow is available at:** https://doi.org/10.5281/zenodo.3271565 ^13^.

**License:** Creative Commons CC BY license.

### Packages used

This workflow depends on various packages from version 3.7 of the Bioconductor project, running on R version 3.5.0 or higher. A complete list of the packages used for this workflow is shown below:


sessionInfo()

R version 3.5.1 (2018-07-02)
Platform: x86_64-w64-mingw32/x64 (64-bit)
Running under: Windows 10 x64 (build 17134)

Matrix products: default

locale:
[1] LC_COLLATE=English_United Kingdom.1252
[2] LC_CTYPE=English_United Kingdom.1252
[3] LC_MONETARY=English_United Kingdom.1252
[4] LC_NUMERIC=C
[5] LC_TIME=English_United Kingdom.1252

attached base packages:
[1] parallel stats4 stats graphics grDevices utils datasets
[8] methods base

other attached packages:
[1] airway_1.2.0 SnowballC_0.5.1
[3] tm_0.7-5 NLP_0.2-0
[5] wordcloud_2.6 org.Hs.eg.db_3.7.0
[7] AnnotationDbi_1.44.0 limma_3.38.2
[9] DESeq2_1.22.1 SummarizedExperiment_1.12.0
[11] DelayedArray_0.8.0 BiocParallel_1.16.1
[13] matrixStats_0.54.0 Biobase_2.42.0
[15] GenomicRanges_1.34.0 GenomeInfoDb_1.18.1
[17] IRanges_2.16.0 S4Vectors_0.20.1
[19] BiocGenerics_0.28.0 igraph_1.2.2
[21] GGally_1.4.0 kableExtra_0.9.0
[23] knitr_1.20 reshape2_1.4.3
[25] ggrepel_0.8.0 cowplot_0.9.3
[27] gplots_3.0.1 ggplot2_3.1.0
[29] RColorBrewer_1.1-2

loaded via a namespace (and not attached):
[1] colorspace_1.3-2 rprojroot_1.3-2
[3] htmlTable_1.12 XVector_0.22.0
[5] base64enc_0.1-3 fs_1.2.6
[7] rstudioapi_0.8 remotes_2.0.2
[9] bit64_0.9-7 xml2_1.2.0
[11] codetools_0.2-15 splines_3.5.1
[13] geneplotter_1.60.0 pkgload_1.0.2
[15] Formula_1.2-3 annotate_1.60.0
[17] cluster_2.0.7-1 intergraph_2.0-2
[19] readr_1.2.1 compiler_3.5.1
[21] httr_1.3.1 backports_1.1.2
[23] assertthat_0.2.0 Matrix_1.2-15
[25] lazyeval_0.2.1 cli_1.0.1
[27] acepack_1.4.1 htmltools_0.3.6
[29] prettyunits_1.0.2 tools_3.5.1
[31] bindrcpp_0.2.2 coda_0.19-2
[33] gtable_0.2.0 glue_1.3.0
[35] GenomeInfoDbData_1.2.0 dplyr_0.7.8
[37] BiocWorkflowTools_1.8.0 Rcpp_1.0.0
[39] slam_0.1-43 statnet.common_4.1.4
[41] gdata_2.18.0 xfun_0.4
[43] stringr_1.3.1 network_1.13.0.1
[45] ps_1.2.1 testthat_2.0.1
[47] rvest_0.3.2 gtools_3.8.1
[49] devtools_2.0.1 XML_3.98-1.16
[51] zlibbioc_1.28.0 scales_1.0.0
[53] hms_0.4.2 yaml_2.2.0
[55] memoise_1.1.0 gridExtra_2.3
[57] rpart_4.1-13 RSQLite_2.1.1
[59] reshape_0.8.8 latticeExtra_0.6-28
[61] stringi_1.2.4 genefilter_1.64.0
[63] desc_1.2.0 checkmate_1.8.5
[65] caTools_1.17.1.1 pkgbuild_1.0.2
[67] rlang_0.3.0.1 pkgconfig_2.0.2
[69] bitops_1.0-6 evaluate_0.12
[71] lattice_0.20-38 purrr_0.2.5
[73] bindr_0.1.1 htmlwidgets_1.3
[75] bit_1.1-14 processx_3.2.0
[77] tidyselect_0.2.5 plyr_1.8.4
[79] magrittr_1.5 bookdown_0.7
[81] R6_2.3.0 Hmisc_4.1-1
[83] sna_2.4 DBI_1.0.0
[85] pillar_1.3.0 foreign_0.8-71
[87] withr_2.1.2 survival_2.43-3
[89] RCurl_1.95-4.11 nnet_7.3-12
[91] tibble_1.4.2 crayon_1.3.4
[93] KernSmooth_2.23-15 rmarkdown_1.10
[95] usethis_1.4.0 locfit_1.5-9.1
[97] grid_3.5.1 data.table_1.11.8
[99] blob_1.1.1 callr_3.0.0
[101] git2r_0.23.0 digest_0.6.18
[103] xtable_1.8-3 munsell_0.5.0
[105] viridisLite_0.3.0 sessioninfo_1.1.1

