# Supplementary material for: Enhancing gene set enrichment using networks
Source: F1000Res. 2019 Jul 16;8:129. Originally published 2019 Jan 30. [Version 2] doi: 10.12688/f1000research.17824.2 (PMC6446501; doi:10.12688/f1000research.17824.2)
Supplement: Peer review history

# Peer review: Reviewer response for version 2

**DOI:** 10.5256/f1000research.21861.r51269

**Published:** 2019-10-02

**Authors:** Jill P. Mesirov, Alexander Wenzel

**Affiliations:** 1 Institute of Genomic Medicine, University of California, San Diego, San Diego, CA, USA; 2 University of California, San Diego, San Diego, CA, USA

Regretfully, many of our previous comments have not been adequately addressed in the current revision and the author’s response, particularly in regard to comparing the proposed method to existing work and justifying its ability to prioritize gene sets in a biologically relevant way. We also have concerns about design decisions in the algorithm and workflow, including the choice of Jaccard cut off and the use of categorical enrichment scoring, that are not adequately explained in the manuscript and responses and continuing technical problems in running the code. 

1. The author acknowledges the similarity to the Enrichment Map method (Merico et al. 2010 ^1^) and notes that it is cited in the manuscript. However, the method is never explicitly mentioned in the text nor is its similarity to the author’s method mentioned even though the similarity is mentioned in the response to review. Given this similarity we feel the text should include a comparison or a discussion of benefits or drawbacks of the respective methods. While the author responded that Merico et al. ^1^ is cited, the citation only refers to the use of the Jaccard method. The response to review contains the following sentence, “The present workflow implements a similar functionality in R and allows for integration in automated analysis pipelines.” Why not just edit or expand on this, specifying that it refers to Enrichment Map, and insert into main text? 
2. The author has not clarified whether “umbrella” is meant as a synonym for hierarchy or if it is describing some other structure. As the author does note that a collection need not have an explicitly defined hierarchy for this method to be useful, how would a potential user determine if such an “umbrella” structure was present and whether or not a given gene set collection would be a good candidate for this approach? 
3. In discussing the Jaccard cut off, the author mentions the general properties preferred in an optimal network, but it is still not clear how a user would robustly evaluate their choice of Jaccard coefficient. Is it sufficient to simply assure that there are at least two distinct components in the graph or is another heuristic more appropriate? Again, some indication of quantitation would be helpful to the user. 
4. The author notes that additional validation with respect to different parameter settings and input datasets is planned for future work. We are not clear why the author cannot indicate how the results in Figure 5 recapitulate known biology in the airway dataset or if they would help the user draw new conclusions. It is unclear from the manuscript whether or not these clusters are in fact useful for further biological interpretation downstream of the proposed method. Wouldn’t this be the main point of using such a method? 
5. With regard to the choice of gene set collection (Hallmarks, KEGG, Reactome, etc.), we would encourage discussion as to the merits or drawbacks of different collections. We note that the Hallmark collection is the most frequently accessed gene set collection in MSigDB and was built using a method that included, in part, clustering of existing gene sets. The paper could be significantly strengthened by comparison of the results of the gene set clustering and prioritization based on enrichment scores in RICHNET to the existing Hallmark collection. We also note that collections of gene sets used to create each individual Hallmark gene set are available on MSigDB. 
6. The author notes that a continuous coloring scheme for output visualizations could be developed using the negative log10 of the p-values. This is certainly possible, but the author has not addressed our concerns involving the coarse, discrete categorization of gene sets as “Up” or “Down” when many popular algorithms for calculating continuous enrichment scores exist. This design decision should be justified in a bit more detail. 
7. Technical problems in running the code: The author has fixed the original bugs in the analysis. However, we encountered the following technical issues when running the new version of the R markdown notebook: a.The EGSEA package is not included in the setup code. Thus, if a user did not have it previously installed, then an error would be triggered later in the analysis. b. The R markdown notebook itself as accessed from the DOI contains notes to comment or delete code “for real submission”. We are therefore uncertain if we are running the correct code for this analysis. c. We encountered the error “Error in `diag<-`(`*tmp*`,value=0): only matrix diagonals can be replace” at line 185 of the notebook and were unable to continue with the analysis. There is currently a high burden on the users of this code to identify and solve package dependencies and other errors whose origins are not immediately clear to those not familiar with the underlying packages. These concerns should be addressed to make the workflow distribution more robust and user-friendly. 

We confirm that we have read this submission and believe that we have an appropriate level of expertise to confirm that it is of an acceptable scientific standard, however we have significant reservations, as outlined above.

## References

[1] : Enrichment map: a network-based method for gene-set enrichment visualization and interpretation. *PLoS One*.2010;5(11) : 10.1371/journal.pone.0013984 e13984 10.1371/journal.pone.0013984 21085593PMC2981572

# Peer review: Reviewer response for version 1

**DOI:** 10.5256/f1000research.19488.r45393

**Published:** 2019-04-10

**Authors:** Rita Casadio

**Affiliations:** 1 Biocomputing Group, CIG, Interdepartmental Center «Luigi Galvani» for Integrated Studies of Bioinformatics, Biophysics and Biocomplexity, University of Bologna, Bologna, Italy

The authors present an efficient and very useful method for gene set analysis. Their approach is network based and facilitates retrieval of sets of genes that are highly connected. Grouping is due to common context (molecular pathways, biological function, tissue localisation). Furthermore, this procedure facilitates gene enrichment and the identification of singletons or poorly connected islands.

 The RICHNET workflow stands as a user friendly workflow that can be easily incorporated in automated analysis pipelines.

I confirm that I have read this submission and believe that I have an appropriate level of expertise to confirm that it is of an acceptable scientific standard.

# Response

**Published:** 2019-07-09

**Authors:** Michael Prummer

**Affiliations:** NEXUS Personalized Health Technologies, ETH Zurich, Switzerland

*Thank you for reviewing the manuscript and for your very positive comments.*

# Peer review: Reviewer response for version 1

**DOI:** 10.5256/f1000research.19488.r45391

**Published:** 2019-04-02

**Authors:** Jill P. Mesirov, Alexander Wenzel

**Affiliations:** 1 Institute of Genomic Medicine, University of California, San Diego, San Diego, CA, USA; 2 University of California, San Diego, San Diego, CA, USA

While the author does address an important general issue of downstream analysis of gene set enrichment results, his approach is almost identical to “ *Enrichment Map: A Network-Based Method for Gene-Set Enrichment Visualization and Interpretation” *(Merico *et al.*, 2010 ^1^). Furthermore, Enrichment Map provides much more information in its visualization including the size of the gene set (via the size of the node); the level of enrichment of the gene sets (via a color gradient from blue for down- to red for up-regulation); as well as the extent of overlap between gene sets (via the thickness of the edge connecting gene sets using the Jaccard index as a weight). Even considering the manuscript independent of this issue, there are a number of other concerns with the study (some minor but some more major) that require attention from the author and a major revision of the manuscript. They are listed more or less in the order that the issue appears in the manuscript.

1. The author claims that gene set analysis “often results in finding hundreds of differentially regulated pathways”. What is the basis for this claim? While it is true that this may occur – it is also true that there may also be no statistically significantly differentially regulated pathways. Has he done experiments on a large number of benchmark data sets to establish this claim of “often”? Moreover, this may be a result of the particular gene set collection used for the analysis. Is it a function of the particular data set or enrichment approach he is using (CAMERA)? We note here that the author never describes the measure CAMERA uses for enrichment nor how up/down is determined. All these open issues should be addressed.
2. The use of “umbrella” organization is somewhat unclear – do you mean hierarchy? Note also the need for a hierarchy of the significantly activated or repressed pathways may result from the author’s choice of gene set collection, which may not provide a significant or specific enough result. This point should be addressed.
3. The author provides little explanation for the arbitrary choice of Jaccard Index > 0.2 as the connectivity threshold. Additionally, as the edges in the networks produced by this method do not represent any other kind of continuous data, has the author considered weighting the edges by the Jaccard index in the visualization to allow the user to see the effect of various thresholds? In fact, Enrichment Map (cited above) uses the Jaccard Index itself to determine the weight of the line connecting two overlapping gene sets. These approaches need to be compared and the choice of threshold better explained and justified.
4. Prioritizing and ranking the active pathways/gene sets by number of gene sets in a network hub and degree of connectivity seems inadequate if one is looking for biological insights. A biological measure of prioritization would be preferable which includes the levels of activation. Often a study is not done in a vacuum and so in fact some signals may be expected – this may serve as an additional measure for prioritization and even validation of the method. This issue is not addressed and should be and some validation of the results of the only test set analysed should be supplied.
5. It is important to include and describe the numerous network-based enrichment methods that have been published and specifically Enrichment Map (cited above) which is a plugin to both Cytoscape and GSEA and does exactly what the author is describing (downstream analysis of gene set enrichment results) but is never mentioned or referenced. At the very least its performance should be compared to the author’s method. This is a requisite for any newly proposed method.
6. The goal of this method is to “focus is on supporting scientists in result interpretation by bringing order into the list of differentially regulated gene sets based on biological rather than pure statistical arguments.” But one might ask why do this based on network measures of association and the visualization is very sparse in what it represents as noted above. Also, if this is the goal – it is incumbent on the author to show why this works better than other existing methods (see above) or even using a more sensitive/specific collection of gene sets, e.g., the Hallmark collection in MSigDB (Liberzon *et al.*, 2015 ^2^). This provides a collection of sets for which essentially this work has been done with additional biological curation. Comparison with other methods and with the use of other gene set collections should be included.
7. The author’s example on the “airway” data set employs an analysis with KEGG, Reactome, and BioCarta. Using these 3 databases together means there will be a large amount of redundancy and overlap in the gene sets he uses – again it would be important to compare his results using these collections against the Hallmark collection. Furthermore – why just the results from one data set? The method would be better tested against multiple data sets – some where the signal is very strong and some where the signal is weaker.
8. The author uses the CAMERA method for testing gene sets. This method produces only a binary “Up” or “Down” measurement of enrichment which is used to color the nodes in the resulting network visualizations. This is a very coarse way of testing gene sets. The interpretability of the network visualizations could be improved by using a method such as GSEA, which gives a continuous enrichment score, and coloring the nodes with a gradient to compare degrees of up- or down-regulation and weighting the edges as is done in Enrichment map as noted above.
9. Finally – after application of RICHNET the author only describes and discusses the nature of his resulting networks. There is no discussion that we could see of the biological insights gained, how realistic they were, whether they recapitulated known signals in the data set, etc.
10. **Technical concerns with the code as presented: **The analysis fails with an unintuitive error (“could not coerce net to a network object”) if the library “Intergraph” is not installed. While this library is listed in the sessionInfo() printout in the manuscript, this library should be included in the first library() cell of the notebook since its absence is not immediately obvious given this error.

We confirm that we have read this submission and believe that we have an appropriate level of expertise to confirm that it is of an acceptable scientific standard, however we have significant reservations, as outlined above.

## References

[1] : Enrichment map: a network-based method for gene-set enrichment visualization and interpretation. *PLoS One*.2010;5(11) : 10.1371/journal.pone.0013984 e13984 10.1371/journal.pone.0013984 21085593PMC2981572

[2] : The Molecular Signatures Database (MSigDB) hallmark gene set collection. *Cell Syst*.2015;1(6) : 10.1016/j.cels.2015.12.004 417-425 10.1016/j.cels.2015.12.004 26771021PMC4707969

# Response

**Published:** 2019-07-08

**Authors:** Michael Prummer

**Affiliations:** NEXUS Personalized Health Technologies, ETH Zurich, Switzerland

*Thank you for reviewing our manuscript and for your critical comments. Below are point-by-point responses to the individual comments.*

The remark on the similarity of this work with prior work by others is fully justified and is discussed in the manuscript. The mentioned work by Merico *et al.* is included as reference 6.

The open nature of this workflow script allows for a straightforward implementation of any additional graphical feature should the user of this workflow wish to do so.

By using the term "often" I was hoping to emphasize that while the statement is true for more than a small number of cases there may be cases where the statement is wrong. How often the statement is true or whether it depends on the choice of geneset or enrichment algorithm or FDR cutoff is less important as long as there exist enough cases for which the workflow presented here may be useful.

Most geneset collections consist of largely overlapping sets which describe very similar processes or properties. The workflow presented here was made for such cases. For the workflow to be useful, an explicit hierarchy, such as with GO-terms, is not required.

The network as constructed here has indeed edges weighted by the Jaccard index. Visualization by varying edge thickness has proven to be difficult to discriminate in my hands. The open nature of this workflow script should allow for an easy implementation of this feature should a user wish to do so.

To address the choice of the Jaccard index cutoff the following sentence has been added at the end of section **Network construction**: “As a guide for finding a reasonable threshold a broad distribution of disjoint cluster sizes is desired. Network analysis does not help if the cutoff is too large (no connections) or too small (all sets are connected with each other).”

One measure of quality of the network construction and community detection is the semantic purity of the clusters. This can be easily seen for the binary systems (and is discussed there) and to a lesser extent for the larger clusters. Whether biological insight can be gained depends strongly on the choice of the genesets in relation to the field of study. RICHNET provides a means of grouping and organizing results and does not generate them.

Enrichment Map is indeed very similar and a perfectly fine interactive tool (see reference 6 of the manuscript). The present workflow implements a similar functionality in R and allows for integration in automated analysis pipelines.

Many of our clients find the hallmark geneset too generic and prefer more diverse geneset collections, such as, KEGG or Reactome.

Your suggestion to include more test results with respect to different geneset libraries, Jacquard score cutoffs, community detection algorithms, and datasets is well received. We are planning to do this in the future.

Using the negative log of the enrichment p-value as enrichment score has been demonstrated in numerous cases in the literature and replacing the categorical colour scale by a continuous one is possible.

Biological interpretation of the geneset clusters produced here is beyond the scope of this work.

The package **intergraph **was included in the library installation and loading part.

# Peer review: Reviewer response for version 1

**DOI:** 10.5256/f1000research.19488.r44250

**Published:** 2019-03-13

**Authors:** Monther Alhamdoosh

**Affiliations:** 1 CSL Limited, Parkville, Victoria, Australia; 2 Bio21 Institute, The University of Melbourne, Parkville, Victoria, Australia

**Summary**

 This workflow paper proposes an interesting approach for prioritizing gene sets in gene set analysis by utilizing network-based algorithms. The author presented a supposedly working code that is reproducible. They proposed focusing on communities of gene sets in order to identify gene sets that are more relevant to the conditions under study. Nonetheless, they show how to explore singletons and binary systems in the list of significant gene sets. Moreover, natural language processing methods were used to annotate the clusters of gene sets. I found this particular extension very useful to summarize gene sets analysis results. However, I didn't get that far in running the code. Please see my comments below for improvements. 

**Major comments**

- Add code to install pre-requisite packages. In my case I had to run the following command to obtain missing packages:

install.packages(c("cowplot", "ggrepel", "kableExtra", "igraph", "GGally", "wordcloud", "tm", "SnowballC"))

- Add code to install the airway experiment package BiocManager::install("airway", version = "3.8") 
- I managed to reproduce the analysis up to the point of generating the first network. First, it was required to install the c("network", "sna", "scales") packages, which was not explained in the text. Then, error raised while invoking ggnet2: 

Error in ggnet2(net, size = 2, color = "Direction", palette = palette, : could not coerce net to a network object

  

- Not sure whether related to the version of R. This is my R session: 

R version 3.5.0 (2018-04-23)

 Platform: x86_64-pc-linux-gnu (64-bit)

 Running under: RHEL

 Matrix products: default

 BLAS:****ps/r/3.5.0/lib64/R/lib/libRblas.so

 LAPACK: ****/apps/r/3.5.0/lib64/R/lib/libRlapack.so

 locale:

  [1] LC_CTYPE=en_US.UTF-8       LC_NUMERIC=C               LC_TIME=en_US.UTF-8       

  [4] LC_COLLATE=en_US.UTF-8     LC_MONETARY=en_US.UTF-8    LC_MESSAGES=en_US.UTF-8   

  [7] LC_PAPER=en_US.UTF-8       LC_NAME=C                  LC_ADDRESS=C              

 [10] LC_TELEPHONE=C             LC_MEASUREMENT=en_US.UTF-8 LC_IDENTIFICATION=C       

 attached base packages:

 [1] parallel  stats4    stats     graphics  grDevices utils     datasets  methods   base     

 other attached packages:

  [1] airway_1.2.0                SnowballC_0.6.0             tm_0.7-6                   

  [4] NLP_0.2-0                   wordcloud_2.6               org.Hs.eg.db_3.7.0         

  [7] AnnotationDbi_1.44.0        limma_3.38.2                DESeq2_1.22.1              

 [10] SummarizedExperiment_1.12.0 DelayedArray_0.8.0          BiocParallel_1.16.0        

 [13] matrixStats_0.54.0          Biobase_2.42.0              GenomicRanges_1.34.0       

 [16] GenomeInfoDb_1.18.1         IRanges_2.16.0              S4Vectors_0.20.1           

 [19] BiocGenerics_0.28.0         GGally_1.4.0                igraph_1.2.4               

 [22] kableExtra_1.0.1            knitr_1.20                  reshape2_1.4.3             

 [25] ggrepel_0.8.0               cowplot_0.9.4               gplots_3.0.3               

 [28] ggplot2_3.1.0               RColorBrewer_1.1-2         

 loaded via a namespace (and not attached):

  [1] bitops_1.0-6           bit64_0.9-8            webshot_0.5.1          httr_1.3.1            

  [5] rprojroot_1.3-2        tools_3.5.0            backports_1.1.2        R6_2.3.0              

  [9] rpart_4.1-13           KernSmooth_2.23-15     Hmisc_4.1-1            DBI_1.0.0             

 [13] lazyeval_0.2.1         colorspace_1.4-0       nnet_7.3-12            withr_2.1.2           

 [17] tidyselect_0.2.5       gridExtra_2.3          bit_1.1-14             compiler_3.5.0        

 [21] rvest_0.3.2            htmlTable_1.12         network_1.14-377       xml2_1.2.0            

 [25] slam_0.1-45            caTools_1.17.1.1       scales_1.0.0           checkmate_1.8.5       

 [29] readr_1.1.1            genefilter_1.64.0      stringr_1.3.1          digest_0.6.18         

 [33] foreign_0.8-71         rmarkdown_1.10         XVector_0.22.0         base64enc_0.1-4       

 [37] pkgconfig_2.0.2        htmltools_0.3.6        htmlwidgets_1.3        rlang_0.3.0.1         

 [41] rstudioapi_0.8         RSQLite_2.1.1          bindr_0.1.1            statnet.common_4.2.0  

 [45] gtools_3.8.1           acepack_1.4.1          dplyr_0.7.8            RCurl_1.96-0          

 [49] magrittr_1.5           GenomeInfoDbData_1.2.0 Formula_1.2-3          Matrix_1.2-15         

 [53] Rcpp_1.0.0             munsell_0.5.0          stringi_1.2.4          yaml_2.2.0            

 [57] zlibbioc_1.28.0        plyr_1.8.4             grid_3.5.0             blob_1.1.1            

 [61] gdata_2.18.0           crayon_1.3.4           lattice_0.20-38        splines_3.5.0         

 [65] annotate_1.60.0        hms_0.4.2              sna_2.4                locfit_1.5-9.1        

 [69] pillar_1.3.0           geneplotter_1.60.0     XML_3.99-0             glue_1.3.0            

 [73] evaluate_0.12          latticeExtra_0.6-28    data.table_1.11.8      gtable_0.2.0          

 [77] purrr_0.2.5            reshape_0.8.8          assertthat_0.2.0       xtable_1.8-3          

 [81] coda_0.19-2            viridisLite_0.3.0      survival_2.43-1        tibble_1.4.2          

 [85] memoise_1.1.0          bindrcpp_0.2.2         cluster_2.0.7-1 

- There are 58 gene sets that are significant in both the uni-directional and bi-directional tests. The former was assumed to be the determinant of the direction of the gene set, which is a bit confusing here. It would be good if the author could elaborate on the reasoning behind assigning the directional status.
- It would be good to highlight the relationship between the identified clusters and annotations, and the underlying experimental conditions. 

**Minor comments**

- EGSEAdata experiment package can be used to load huge number of gene sets including MSigDB gene sets. This is an example:

library(EGSEAdata)

 library(EGSEA)

 gset = buildMSigDBIdx(entrezIDs = res$entrezgene, species = "human", geneSets = "c2", min.size = 3)

 idx = gset$c2@idx

 gs.libs = sapply(names(idx), function(x) strsplit(x, "_")[[1]][1])

 idx = idx[which(gs.libs %in% c("KEGG", "REACTOME", "BIOCARTA"))]

 Please see this workflow paper for more information ^1^. 

- The workflow is tailored towards the camera analysis. What if users want to use this workflow for other GSA methods? A more generic example is needed. 
- Any possibility to wrap the network analysis code and make it easier for users to invoke as a function? Probably, an R package named RICHNET can be developed along with this wonderful workflow. 

I confirm that I have read this submission and believe that I have an appropriate level of expertise to confirm that it is of an acceptable scientific standard, however I have significant reservations, as outlined above.

## References

[1] : Easy and efficient ensemble gene set testing with EGSEA. *F1000Res*.2017;6: 10.12688/f1000research.12544.1 2010 10.12688/f1000research.12544.1 29333246PMC5747338

# Response

**Published:** 2019-07-08

**Authors:** Michael Prummer

**Affiliations:** NEXUS Personalized Health Technologies, ETH Zurich, Switzerland

*Thank you for reviewing our manuscript and for your constructive comments. Below are point-by-point responses to the individual comments.*

 The missing packages were included in the initial package installation and loading part.

 The source of the error in ggnet2 is fixed.

 Uni- and bi-directional tests are preformed in parallel. Afterwards, priority is given to the uni-directional test as it is more stringent (either up- or down-regulated genes). The bi-directional case is biologically meaningful as well as an upregulation of and inhibitory member of a pathway has the same effect as the downregulation of an activatory member. But all these details are related to one particular choice of enrichment analysis whereas the extent of this work starts after a list of candidate genesets was generated by any appropriate method. Biological interpretation of the geneset clustering results produced here is beyond the scope of this work.

 A code chunk using EGSEAdata to build the geneset library was included in the manuscript.

 The following sentence is included in paragraph 2 of the discussion: “One advantage over other approaches is that it does not depend on a particular geneset library or geneset analysis method. Any means of selecting genesets of interest can be used.”

# Peer review: Reviewer response for version 1

**DOI:** 10.5256/f1000research.19488.r44249

**Published:** 2019-03-07

**Authors:** Kimberly Glass

**Affiliations:** 1 Department of Medicine, Harvard Medical School, Boston, MA, USA; 2 Department of Biostatistics, Harvard T.H. Chan School of Public Health, Harvard University, Boston, MA, USA

In "Enhancing gene set enrichment using networks" the author describes a pipeline to visualize the gene sets associated with a particular differential-expression analysis as a network. In this network connections between gene sets are based on common/shared gene annotations. The paper is clearly written and the decisions made in the pipeline are reasonable. There are only a few points on which I would like to see more discussion:

1. In the introduction the authors mention the Jaccard index as a similarity measure (alongside coexpression of genes in WGCNA). There are many similarity measures: hamming distance, cosine similarity, Fisher's exact test, as well as many other measures for continuous variables that can easily be adapted to binary variables. Which one of these is used to construct the gene-set network could impact the structure of the network. The pros and cons of using the Jaccard index as well as other similarity measures warrants more discussion (e.g. some may better capture relationships between terms with many gene annotations, and some may better capture relationships between terms with fewer gene annotations).
2. GO terms are structured as a DAG, with genes annotated to child terms propagating to parent terms. This underlying structure will impact the structure of the similarity network between GO terms, and is worth pointing out in the manuscript.
3. In the introduction, the authors state "the clusters can be categorized as....medium and large or dense and loose clusters". The author should either include more discussion about how these can be quantified (i.e. what is a "medium" cluster) or they should illustrate the quantification of these categories in their example.
4. The equation “J=NumberofcommongenesNumberofallgenes” looks mis-formatted. It also would be better presented as: “J = intersect(set A, set B)/union(set A, set B)”.
5. The authors should also consider the structure of a general gene-set network (one not restricted to gene-sets associated with differentially-expressed genes). It is possible that the singletons/doublets that the authors remove may simply come from a sparser area of this "general" gene-set network (and the clusters a denser area), in which case the pruning step is removing relevant results (while retaining less informative ones that might be picked up by chance).
6. In naming the clusters, I would suggest normalizing the number of instances of a word against its frequency in the entire database. For example "cell" is a much more common word in KEGG/GO term names than "glycolosis". From a biological point of view, if all the terms that contain "glycolosis" are in the same cluster (even if it’s only 1-2 terms), this is likely much more interesting to highlight than if "cell" appears frequently in that cluster (but also in many other terms outside of the cluster).
7. Do the authors have any thoughts about how to interpret clusters with "missing titles" (no word appearing more than once)?

Minor comments:

- Be sure to spell out the GO and KEGG acronym for first usage.
- Some of the longer names in Figure 5 appear truncated (e.g. "cell death signalling via").

I confirm that I have read this submission and believe that I have an appropriate level of expertise to confirm that it is of an acceptable scientific standard.

# Response

**Published:** 2019-07-08

**Authors:** Michael Prummer

**Affiliations:** NEXUS Personalized Health Technologies, ETH Zurich, Switzerland

*Thank you for reviewing our manuscript and for your constructive comments. Below are point-by-point responses to the individual comments.*

 While a discussion on the influence of different distance metrics on network structure is interesting it is not in the scope of this work. Sidenote: a discussion on the influence of different community detection algorithms would be interesting as well. These aspects are critical to any attempt at clustering data but it is assumed here that the qualified reader and user of this workflow is aware of it. The issue is briefly mentioned in paragraph four of the discussion.

 Avoiding the use of GO terms was an attempt to avoid having to open up the discussion about the influence of their hierarchical structure on results. I am convinced this would make the discussion unnecessarily complicated and that it is better done elsewhere.

 The equation defining the Jaccard index is formatted correctly in the PDF.

 In the manuscript it is emphasized that nothing is removed or deemed irrelevant and that putting aside singletons and doublets is just a means of sorting. Indeed, an unusually large proportion of singletons may indicate an unexplored area of biology. In such a situation, relying on common knowledge in the form of published genesets may not be the wisest way to go at all.

 There are a number of different possibilities to obtain a representative label for a cluster and in this manuscript a relatively simple one was chosen. It may not be the most sophisticated but it is straight forward to understand.

 The following text is included in section **Lattice of annotated networks** on *missing titles*: “This may be indicative for a semantically mixed cluster or for sparse prior knowledge.”
